# Full-length single-molecule sequencing uncovers novel insight into the global landscape of the cold stress response in trifoliate orange (*Citrus trifoliata*)

**DOI:** 10.3389/fpls.2024.1506414

**Published:** 2024-11-18

**Authors:** Yue Wang, Tian Fang, Jihong Liu

**Affiliations:** National Key Laboratory for Germplasm Innovation & Utilization of Horticultural Crops, College of Horticulture and Forestry Science, Huazhong Agricultural University, Wuhan, China

**Keywords:** trifoliate orange, cold stress, full-length transcript, RNA-seq, soluble sugars

## Abstract

Trifoliate orange (*Citrus trifoliata* (L.) Raf.) is a cold-hardy citrus species that contributes to citrus production by frequently serving as a rootstock. Nevertheless, the molecular mechanisms underlying cold tolerance in citrus, particularly post-transcriptional regulation, remain largely unidentified. In this study, we constructed a transcriptome map of trifoliate orange subjected to cold stress by integrating full-length single-molecule sequencing and Illumina short-read sequencing. The hybrid sequencing approach yielded a more comprehensive set of full-length transcripts than was previously available from the reference genome. In particular, the high-quality transcripts enabled the detection of extensive alternative splicing (AS), with intron retention (IR) identified as the predominant AS event in trifoliate orange. Transcriptome analysis revealed that genes associated with starch and sucrose metabolism were significantly enriched among the cold-responsive genes. Consistent with these data, soluble sugar content was elevated by the cold treatments. Additionally, the expression of multiple genes encoding enzymes with antioxidant activity, including *PODs* and SODs, was induced, which plays a pivotal role in the mitigation of continuous ROS production. Furthermore, we observed that AS and transcriptional regulation modulate distinct pathways. We also found that the expression of genes encoding key transcription factors (TFs) was highly induced by cold stress and that some of the mRNAs encoding these key TFs were differentially spliced. This dataset provides comprehensive transcriptional and post-transcriptional profiles of the response to cold stress in trifoliate orange that may help to identify genes that contribute to cold tolerance in citrus.

## Introduction

Citrus fruit is the most widely cultivated fruit globally, and it contains a plethora of beneficial bioactive compounds, including polyphenols and flavonoids (Talon et al., 2020). As a typical fruit tree of the tropical and subtropical zones, citrus is particularly susceptible to the adverse effects of cold and freezing temperatures (Dahro et al., 2023). In the early spring period, when citrus seedlings are planted in the field, they frequently experience damage from low temperatures and frost that reduces growth and impairs development, and in some cases, even kills seedlings. Furthermore, frost is also detrimental to citrus flowers and fruits because if temperatures fall below -2°C for 4 h ice crystals nucleate in the extracellular spaces of fruits, which leads to significant reductions in yield (Huang et al., 2011). Therefore, the improvement of cold tolerance has long been an important target for citrus breeding programs. Trifoliate orange [*Citrus trifoliata* (L.) Raf.] is an extremely cold hardy citrus species (Sahin-Çevik, 2013) that is widely used as a rootstock for citrus production (Huang et al., 2021). However, for some citrus species with reproductive barriers, the traditional breeding pipeline, such as hybridization-based breeding, is either time-consuming or impossible (Zhang et al., 2018). As an alternative, genetic engineering has proven to be an effective and efficient approach to creating cold-tolerant transgenic plants. The elucidation of mechanisms that contribute to cold tolerance and the identification of genes associated with cold hardiness provide a basis for genetic engineering and breeding.

As sessile organisms, plants are unable to migrate to favorable environments. Therefore, plants have evolved a range of strategies to cope with cold stress that involve an array of transcriptional, physiological and biochemical changes (Ding and Yang, 2022). The upregulation of cold-responsive gene expression is critical for cold tolerance. For example, C-repeat binding factor (CBF) is a pivotal transcription factor that directly activates the expression of cold-responsive (COR) genes (Kidokoro et al., 2022). Activation of these genes leads to the accumulation of protective substances, such as osmolytes and cryoprotective proteins (Shi et al., 2018) and therefore, facilitates cold acclimation and freezing tolerance. Despite considerable advances in unraveling the transcriptional network associated with the cold stress response in citrus over the past two decades (Dahro et al., 2023), research into the post-transcriptional regulation of COR genes remains relatively limited.

Alternative splicing (AS) is a post-transcriptional regulatory process that produces multiple mRNA variants from intron-containing genes (Kalsotra and Cooper, 2011; Reddy et al., 2013) and that increases the diversity and complexity of the transcriptome and proteome. AS events can be classified into five categories: intron retention (IR), exon skipping (ES), alternative 3′ splice site selection (A3SS), alternative 5′ splice site selection (A5SS), and mutually exclusive exons (MXEs). IR is the most common form of AS in plants. Emerging evidence indicates that alterations in AS globally influence plant development and stress responses. In *Arabidopsis*, *SME1* plays an integral role in regulating plant development and the response to abiotic stress by modulating the specificity of spliceosomes (Huertas et al., 2019). A pan-transcriptome analysis demonstrated that alternative splicing plays an instrumental role in regulating the cellular responses to cold stress in rice (Zhong et al., 2024). The participation of AS in the response of plants to cold stress has also been observed in maize (Zhang J. et al., 2022), cassava (Li et al., 2020a) and tea (Li et al., 2020b). Nevertheless, there is a paucity of information on the contribution of AS to the response to cold stress in fruit trees, such as citrus.

Currently, PacBio isoform sequencing (Iso-Seq) offers a direct approach to obtain full-length transcript sequences, thereby enabling the accurate annotation of alternative splicing events. By integrating single-molecule long-read isoform sequencing with RNA sequencing, researchers have been able to perform comprehensive investigations into the transcriptomes of plants, including rice (Zhang et al., 2019; Zhong et al., 2024), cotton (Wang et al., 2018a), bamboo (Luo et al., 2019; Wang et al., 2017) and wheat (Wang et al., 2019). In this study, we constructed a transcriptome map of trifoliate orange using the Iso-Seq and RNA-Seq approaches and elucidated a cold response mechanism for a cold-hardy citrus species. Our dataset provides a comprehensive collection of full-length transcripts and provides evidence that substantial changes in AS are a critical component of the cold stress response in plants. Additionally, our transcriptional analysis revealed a notable upregulation in the expression of genes involved in the accumulation of soluble sugars and encoding enzymes with antioxidant activity, which may contribute to the cold tolerance in trifoliate orange. Furthermore, our findings indicate that the regulation of AS and transcription by cold stress affect different metabolic pathways in citrus. This study offers a systematic characterization of the transcriptome in trifoliate orange during cold stress and thereby establishes a robust foundation for the investigation of candidate genes that may contribute to cold tolerance in citrus.

## Materials and methods

### Plant materials and cold treatment

Three-month-old trifoliate orange seedlings grown in a greenhouse at Huazhong Agricultural University were used in this study. The seedlings were subjected to a cold treatment at -2°C for 12 h and 24 h. Seedlings grown in optimal temperatures were used as controls. Phenotypes were observed after 24 h. Prior to phenotypic observations, leaf tissue was collected at 0h, 12 h and 24 h of cold treatment for Illumina RNA-Seq. Three biological replicates were prepared for each time point. The leaves, stems and roots harvested at 0h and 24 h of cold stress were mixed for SMRT sequencing. All the samples were stored at −80°C for further analysis.

### Measurements of electrolyte leakage and chlorophyll fluorescence

Electrolyte leakage (EL) was measured using a conductivity meter as described previously by Dahro et al. (2016). Briefly, the leaves collected before and after cold stress were cut into small pieces and immediately immersed in 15 ml of double-distilled water. The first electronic conductivity of the samples and a water control, designated C1 and CK1, respectively, was assessed using a conductivity meter (DSS‐307, SPSIC). The leaf samples were then incubated for ten min at 100°C in a boiling water bath and then were allowed to cool at room temperature. Subsequently, the second conductivity (C2, CK2) was examined using the same conductivity meter. The EL was then calculated using the following formula: EL% = (C1-CK1)/(C2-CK2) × 100%. Chlorophyll fluorescence was quantified utilizing an imaging-PAM chlorophyll fluorometer (Walz, Effeltrich). *F_v_*/*F_m_* was calculated using the IMAGING WINGEGE software.

### Histochemical staining of reactive oxygen species (ROS)

The *in situ* accumulation of O_2_ **^·-^** and H_2_O_2_ was detected using histochemical staining techniques with nitro blue tetrazolium (NBT) and 3,3’-diaminobenzidine (DAB), respectively, as described previously by Huang et al. (2013). Briefly, the leaves were incubated in freshly prepared solutions of DAB or NBT. The DAB was dissolved in 50 mM potassium phosphate buffer (pH 3.8) at a concentration of 1 mg·ml^-^¹. The NBT solution was prepared using the same buffer, but at a pH of 7.8. After a 12 h incubation at room temperature in the dark, the chlorophyll was removed by boiling the leaves in 75% ethanol. Subsequently, the leaves were photographed.

### Measurement of soluble sugars

Soluble sugar content was determined using gas chromatography (GC), as described previously by Yu et al. (2012) with modifications. One gram of powder was extracted using 80% methanol, and the solution was subsequently heated to 70°Cfor 30 min. Following an extraction period of 90 min and subsequent centrifugation at 4000×*g* for 10 min, the resulting supernatant was collected, transferred to a 10 ml volumetric flask and 0.2 ml of an internal standard (2.5% w/v phenyl-β-D-glucopyranoside, 2.5% w/v methyl-α-D-glucopyranoside) was added. Following agitation, the solution was transferred to a 2 ml centrifuge tube and clarified by centrifugation at 12,000 rpm for 15 min. Subsequently, 0.5 ml of the supernatant was vacuum-dried at 60°C and redissolved in 800 μl hydroxylamine hydrochloride. Two g of hydroxylamine hydrochloride was dissolved in 100 ml pyridine was incubated at 70°C for one h. Subsequently, 400 μl of hexamethyldisilimide (HMDS) and 200 μl of trimethylchlorosilane (TMCS) were added. Subsequently, 500 μl of each sample was subjected to GC-FID analysis in an Agilent 6890B Gas Chromatography System (Agilent Technologies, Palo Alto, CA, USA), which is equipped with a flame ionisation detector. The analysis of soluble sugars was conducted using an HP-5 column (5% phenyl-methylpolysiloxane, 30 m × 25 μm i.d. × 0.1 μm). Three biological replicates were assessed per sample.

### Illumina RNA-seq and PacBio sequencing library construction

Total RNA was extracted using TaKaRa MiniBEST Plant RNA Extraction Kit and treated with RNase-free DNase I (Takara, Dalian, China) according to the provided protocol. For RNA-seq, a total of 2 µg of input RNA was used for the generation of sequencing libraries using the NEBNext Ultra™ RNA Library Prep Kit for Illumina (NEB, USA) in accordance with the manufacturer’s instructions. Index codes were added to the sequences to facilitate their attribution. Subsequently, the mRNA was subjected to sequencing on an Illumina HiSeq 2500 platform at the Novogene Company (Beijing, China). For PacBio sequencing, the SMARTer PCR cDNA Synthesis Kit was employed for the synthesis of cDNA from mixed plant RNA samples, collected prior to and following exposure to cold stress. In preparation for sequencing, sequencing primers were annealed to the libraries and the polymerase was added to the template with the annealed primer. Thereafter, the template with the associated polymerase was bound to MagBeads and sequencing was performed on a PacBio RSII instrument.

### Analysis of PacBio single-molecule long-reads

The SMRT Link software v6.0 (https://www.pacb.com/support/software-downloads/) was used to identify full-length non-chimeric (FLNC) transcripts from PacBio SMRT reads. The FLNCs were further corrected with the Lordec software (Salmela and Rivals, 2014) using Illumina RNA-seq reads. The Iso-seq3 pipeline was then employed to cluster and polish the FLNC sequence, resulting in the generation of high-quality transcripts. The high-quality transcripts were mapped to the reference genome using the GMAP software (Wu and Watanabe, 2015). The full-length transcripts that we were not able to align to the reference were regarded as novel transcripts. AS events were identified using the aforementioned GTF file produced using the Asprofile software (Florea et al., 2013). The rMATS tool (Shen et al., 2014) was employed to detect differential alternative splicing (DAS) events using the RNA-seq data. We annotated the novel transcripts using the Basic Local Alignment Search Tool (BLAST) searches of several databases including NR (NCBI non-redundant protein sequences), GO (Gene Ontology), KOG (euKaryotic Ortholog Groups) and KEGG (Kyoto Encyclopedia of Genes and Genomes).

### RNA-seq sequencing and analysis

The raw data was computer filtered to remove adaptors and poor-quality bases using the fastp software (Chen et al., 2018). Then the clean reads were mapped to the Citrus genome using Hisat2 (Kim et al., 2015). Samtools was subsequently used to transfer the data into the “bam” format. Gene expression was quantified using the FPKM (Fragments Per Kilobase of transcript per Million mapped reads) value, which was calculated using the Feature Counts software (Liao et al., 2014). The identification of differentially expressed genes (DEGs) was conducted using the Deseq2 software (Love et al., 2014), employing a fold change of ≥2 and a false discovery rate (FDR) of ≤0.05 as the criteria for significance. KEGG enrichment analysis of genes was conducted using TBtools (Chen et al., 2020). TFs were identified by performing BLAST searches using amino acid sequences against the Plant Transcription Factor Database (PlnTFDB) (http://plntfdb.bio.uni-potsdam.de/v3.0/). In the case of the TFs that were newly identified in the course of our analysis, the HMMER software was employed to search protein domains. Only proteins that matched exactly with the domains included in the transcription factor database were considered to be TFs.

### Real-time qPCR

Real-time qPCR was performed using the SYBR Premix ExTaq Green PCR Kit (QIAGEN) as recommended by the manufacturer. The reaction solution consisted of 5 μl of 2× SYBR Green PCR master mix, 1 μl of QN ROX reference dye, 1 μmol/L of forward and reverse primers, and 100 ng of cDNA, resulting in a total volume of 10 μl. The reaction program included an incubation at 95°C for 5 min and then 45 cycles of 10 sec at 95°C followed by 20 sec at 60°C. ACTIN was used as an internal reference gene to normalize the samples, and relative gene expression levels were calculated using the 2^‐ΔΔCT^ method as described previously by Livak and Schmittgen (2001). Three technical replicates were analyzed for each sample. The primers used in this study are listed in **Supplementary Table 1**.

### Statistical analysis

Statistical significance was assessed using a Student’s t-tests in Excel. Data were expressed as mean values ± SD (standard deviation) and were based on at least three replicates, *P values <*0.05 were considered to be statistically significant.

## Results

### The phenotype and physiological response of *Citrus trifoliata* to cold stress

**Figure 1:**
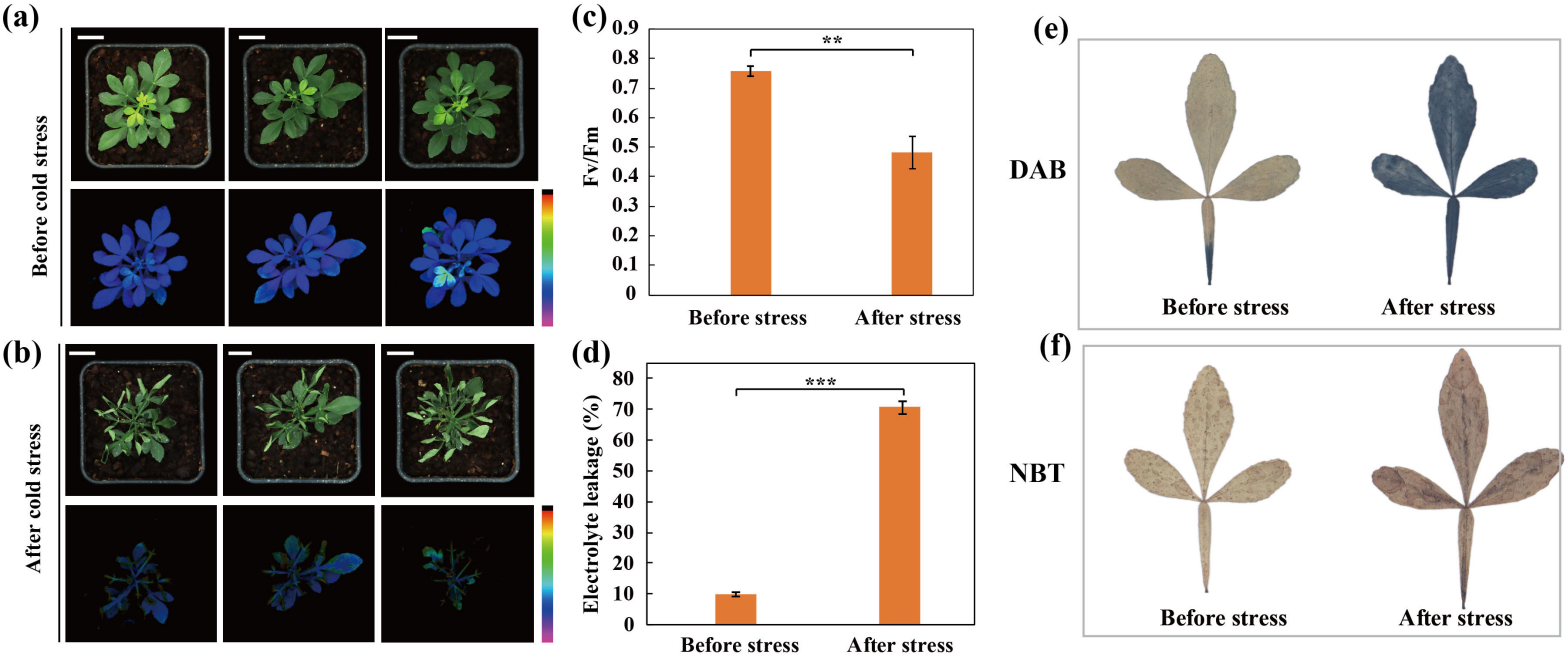
Phenotypes and physiological indices before and after cold stress in trifoliate orange plants. **(A, B)** Phenotype and chlorophyll fluorescence images for trifoliate orange plants before **(A)** and after **(B)** cold stress (Scale bars =1 cm). **(C, D)** *F_v_*/*F_m_* **(C)** and electrolyte leakage **(D)** before and after cold stress. Bars indicate mean values ± SD from three biological replicates. **(E, F)** Histochemical staining with 3,3-diaminobenzidine **(E)** and nitro blue tetrazolium **(F)**.

In order to elucidate the impact of cold stress on trifoliate orange, the seedlings were subjected to a cold treatment at -2°C. Before cold stress, the plants were grown using standard cultivation conditions and exhibited robust growth, as indicated by the intensity of chlorophyll fluorescence, which demonstrated high photosynthetic activity (**Figure 1A**). After cold stress, water soaking and wilting were visually detected in leaves. Moreover, chlorophyll fluorescence was severely reduced in the cold-treated plants (**Figure 1B**). Subsequently, certain physiological indices including electrolyte leakage (EL) levels and maximum quantum efficiency of photosystem II (*F_v_*/*F_m_*) were assessed for the plants before and after the cold treatment (**Figures 1C, D**). In accordance with the phenotypic observations, the electrolyte leakage (EL) levels increased in the leaves during the cold stress treatment and *F_v_*/*F_m_* was significantly reduced following cold stress. Additionally, we observed that the plants accumulated more reactive oxygen species (ROS, H_2_O_2_ and O_2_ **^·-^**) during the cold stress treatment, as revealed by staining with DAB (**Figure 1E**) and NBT (**Figure 1F**), respectively. Taken together, these results indicate that cold stress induces cellular injury and impairs photosynthetic capacity in trifoliate orange.

### Characterization of the full-length transcriptomes in trifoliate orange

**Figure 2:**
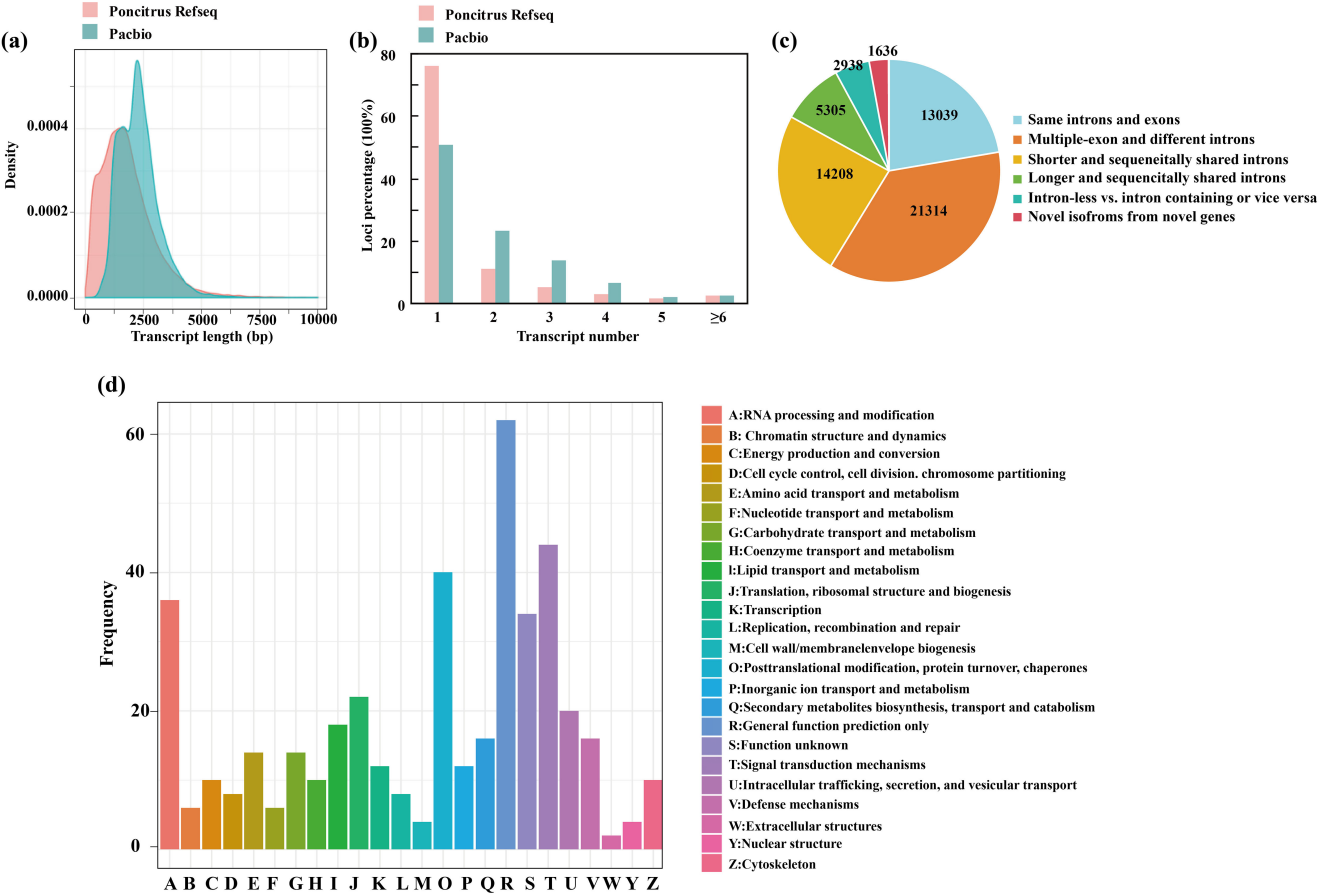
Characterization of full-length transcripts from *C. trifoliata*. **(A–C)** Comparisons of transcript lengths **(A)**, isoform numbers **(B)** and structures **(C)** between the *C. trifoliata* RefSeq annotation and the PacBio data. **(D)** KOG classification of new transcripts.

To obtain high-confidence full-length transcripts in trifoliate orange, total RNA was isolated and mixed from leaves, stems and roots grown in both normal and cold stress conditions (-2°C for 24h) and sequenced on the PacBio SMRT platform. The filtering of adapter and low-quality sequences yielded a total of 27,812,819,951 subreads (**Supplementary Table 2**). Then the SMRT analysis (Gordon et al. 2015) pipeline was used and further corrected with Illumina RNA-seq data using LoRDEC. In total, 767,601 full-length nonchimeric reads (FLNCs) were obtained, with an average length of 2,038. However, the FLNCs usually contain duplicate isoforms. After clustering and polishing using the iso-seq3 pipeline with default parameters, a total of 58,485 high-quality transcripts and 12,341 low-quality transcripts were obtained. The high-quality transcripts were derived from 19,356 gene loci, covering 75.37% of the high-confidence genes annotated in a previous published genome (Huang et al., 2021). The mean length of the transcripts revealed by the Pacbio data were higher than that annotated in trifoliate orange (**Figure 2A**). Of the 19,356 annotated gene loci, 48.95% of the genes had multiple exons (≥2), exceeding the proportion of multiple-exon genes with two or more isoforms in previous annotated genomes (23.90%) (**Figure 2B**). We further compared the transcript structure obtained by us with the previous trifoliate orange annotation. These transcripts were classified into six groups (**Figure 2C**). In total, 1,636 gene loci were identified that did not overlap with any previously annotated genes in the trifoliate orange genome. We then performed a functional characterization of the new transcripts by searching several databases (**Supplementary Table 2**), including NR, GO, KOG and KEGG. According to the KOG analysis, the novel genes were grouped into 24 categories. The top three enriched categories were “[R] for general function prediction”, “[T] Signal transduction mechanisms” and “[O] Posttranslational modification, protein turnover, chaperones” (**Figure 2D**). Only 74 genes were not annotated in any database (**Supplementary Table 3**). In conclusion, the results presented here provide qualified full-length reference sequences based on PacBio sequencing, which are essential for comprehensive profiling of the trifoliate orange transcriptome.

### Alternative splicing (AS) event analysis

**Figure 3:**
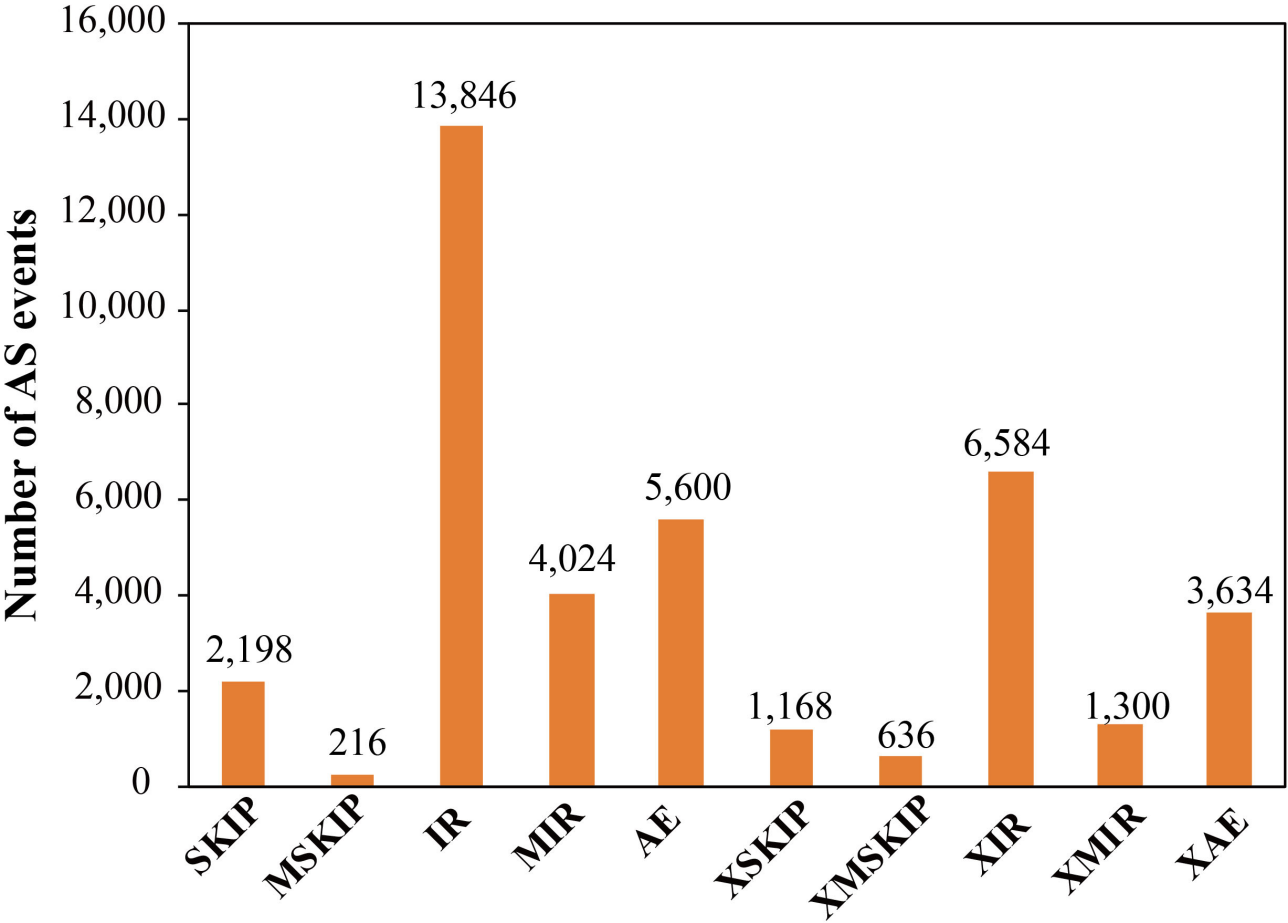
Frequencies of different types of alternative splicing based on Pacbio data.

Some genes produce differentially spliced isoforms of their encoded mRNAs by employing distinct splicing modes, which involves the selection of a variety of different splice sites. This phenomenon is referred to as AS. PacBio long-read sequencing enables the complete sequencing of transcripts. In comparison to second-generation short-read RNA-Seq sequencing, which relies on junction reads for the identification of alternative splicing, third-generation full-length sequencing allows for the direct comparison of full-length sequences, and thus, facilitates the identification of alternative splicing. Here, we identified a total of 39,206 AS events from 12,247 gene loci using the ASprofile software (**Figure 3**). The AS events could be grouped into 10 types, including Exon skipping (SKIP) and cassette exons (MSKIP), retention of a single intron (IR), retention of multiple introns (MIR), alternative exon ends (5’, 3’, or both) (AE), approximate exon skipping (XSKIP) and cassette exons (XMSKIP), approximate retention of single (XIR) and multiple (XMIR) introns, and approximate alternative exon ends (XAE). IR is the primary mode of AS in trifoliate orange, which is consistent with previous work (Wang et al., 2019; Zhang et al., 2019).

### Differentially expression and splicing of genes during cold stress

**Figure 4:**
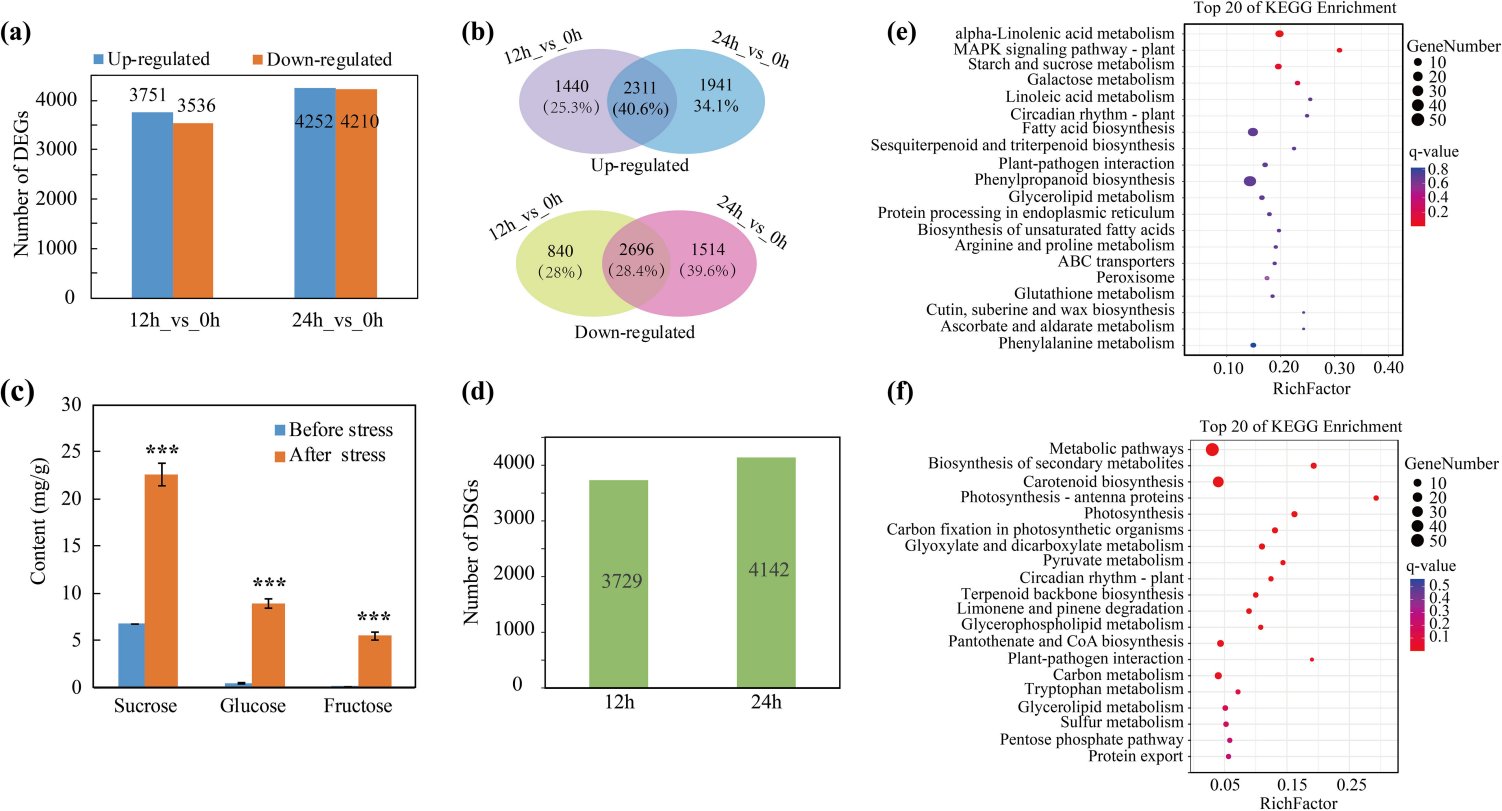
Identification of cold-responsive DEGs and DSGs. **(A)** Numbers of DEGs after cold stress for 12 h and 24 h **(B)** Numbers and percentages of genes up or down regulated at only one or at both time points (12 h and 24 h). **(C)** Quantification of soluble sugars before and after cold stress, ***P < 0.001. **(D)** Number of DSGs after 12 h and 24 h of cold stress. **(E, F)** KEGG enrichment analysis of DEGs and DSGs after cold stress.

To identify the key genes and potential metabolic pathways involved in the response to the cold in trifoliate orange plants, RNA-seq was performed using RNA from control seedlings that were collected prior to the application of cold stress and from seedlings that had been subjected to cold stress for 12 and 24 h. After quality filtration, 5.88 ~ 7.27 Gb of clean reads were obtained for each sample (**Supplementary Table 4**). Then we mapped the high-quality reads to the *C. trifoliata* genome (Huang et al., 2021). We observed mapping rates of ≥ 95.12% for all samples. We calculated the fragments per kilobase per million (FPKM) values for the different RNAs to determine the gene expression levels. The principal component analysis (PCA) indicated good consistency between the biological replicates, thereby providing evidence for the reliability of the RNA-seq data (**Supplementary Figure 1**). Differentially expressed genes were identified before and after cold stress (12 h_vs_0 h and 24 h_vs_0 h) using a false discovery rate (FDR) < 0.05 and |log2fold change|≥ 1 as criteria. A total of 7,287 (3,751 up-regulated genes, 3,536 down-regulated genes) and 8,462 (4,252 up-regulated genes, 4,210 down-regulated genes) DEGs were identified after 12 h and 24 h of cold stress, respectively (**Figure 4A**; **Supplementary Table 5**). A Venn diagram revealed that the expression of 2,311 (40.6%) genes were induced by cold stress after both 12 h and 24 h and that the expression of 2,696 (28.4%) genes were downregulated by cold stress after 12 h and 24 h (**Figure 4B**). To further validate the expression patterns of the DEGs, RT-qPCR was used to quantify the relative expression of several key cold-responsive genes, including *NAC2*, *PAT1*, *CBF1*, *CBF4*, *SOD3* and *INV2* (**Supplementary Figure 2**). The relative quantification was consistent with the transcriptome expression trends and provides reliable evidence for our selection of candidate genes. We then investigated the regulation of AS during cold stress and identified 3,729 and 4,142 differentially spliced genes (DSGs) at 12 h and 24 h, respectively (**Figure 4D**; **Supplementary Table 6**). Notably, we noticed that 3,443 genes that were differentially spliced at 12 h (77.8%) were also differentially spliced at 24 h (**Supplementary Figure 3**), indicating that the regulation of AS is less transient than the regulation of the transcriptome during cold stress.

To gain insight into the pathways regulated by AS and transcription, we conducted a KEGG enrichment analysis for the DEGs and DSGs and found that the regulation of the transcriptome and splicing influence disparate pathways during cold stress. For example, the DEGs were significantly enriched in “alpha-linolenic acid metabolism” and “starch and sucrose metabolism” pathways (**Figure 4E**). Sucrose metabolism has been previously reported to help plants adapt to cold stress (Dahro et al., 2023). Therefore, we further analyzed the expression of the genes associated with the accumulation of sugars. We found that the expression of multiple genes involved in sugar metabolism, such as *SWEETs* (*Sugars Will Eventually be Exported Transporter*), *ERDs* (*Early Responsive to Dehydration*), *SPS* (*Sucrose Phosphate Synthase*), *SS* (*Sucrose Synthase*) and *INV* (*Invertase*), were up-regulated after cold stress (**Supplementary Figure 4**). Soluble sugar content was quantified in the leaves of trifoliate orange plants before and after cold stress. The results demonstrated a notable elevation in the levels of three primary soluble sugars (sucrose, glucose and fructose) following the cold stress treatment (**Figure 4C**). The aforementioned experiment demonstrated that two principal ROS (H_2_O_2_ and O_2_ **^·-^**) accumulated in response to cold stress. Consequently, the expression patterns of genes associated with antioxidants were analyzed. The results indicated that the expression of genes encoding 12 PODs and 4 SODs—two pivotal enzymes that scavenge ROS—were elevated during cold stress (at 12 h or 24 h) relative to the normal conditions (**Supplementary Figure 5**). On the other hand, the DSGs were mainly enriched in “photosynthesis”, “carbon metabolism” and other pathways related basic biological processes (**Figure 4F**). The results indicated that the regulation of AS and the transcriptome differentially influence the cold stress response in citrus. Moreover, the data indicate that the induced expression of genes associated with the accumulation of soluble sugars and the scavenging of ROS may be important for the adaptation of citrus to cold stress.

### Identification of cold-responsive transcription factors in trifoliate orange plants

**Figure 5:**
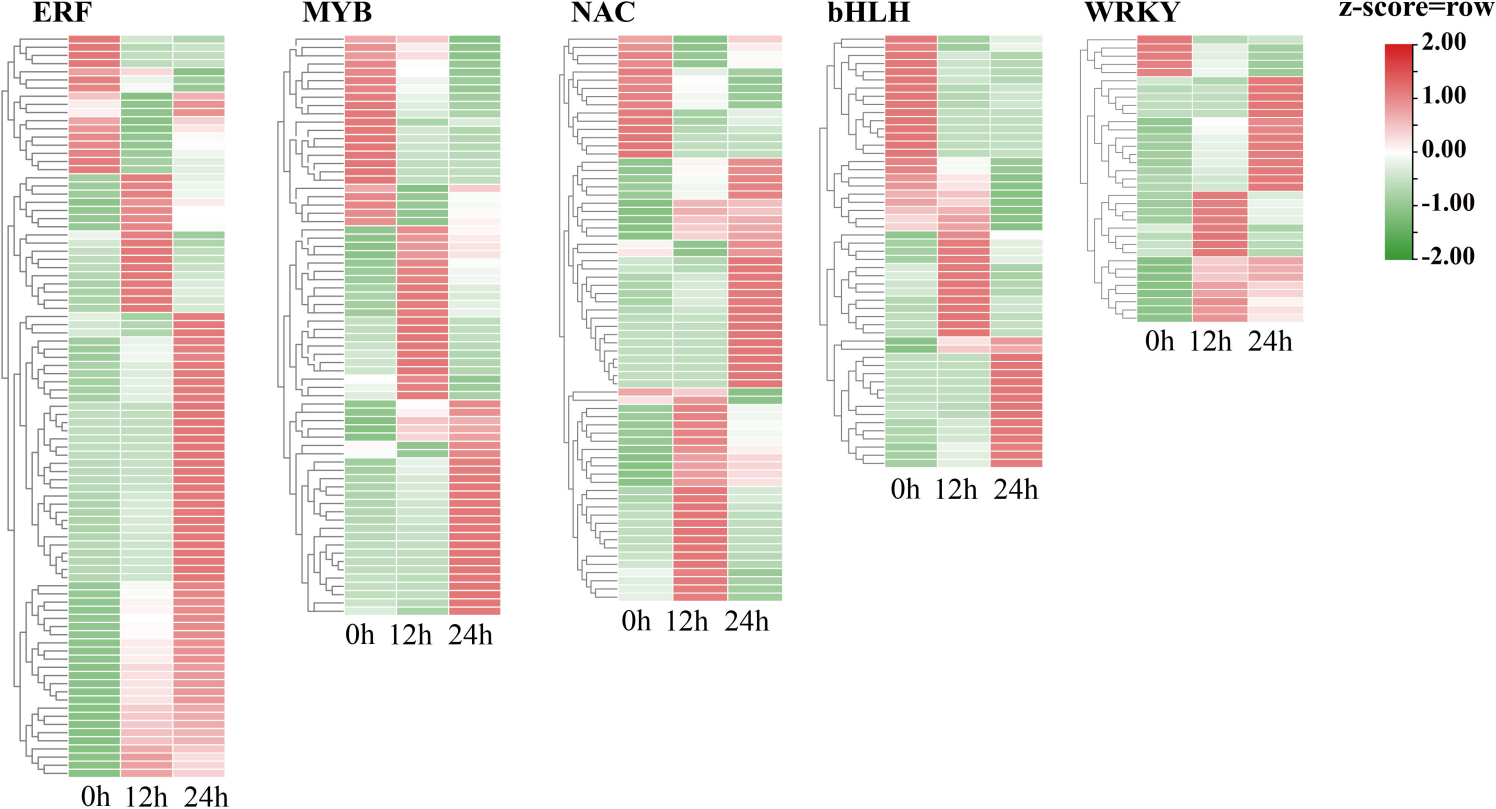
Heatmap showing the expression levels of cold-responsive genes that encode particular types of TFs during cold stress.

Transcription factors play an essential role in the regulation of cold stress responses in plants. To explore the key TFs that regulate cold tolerance in trifoliate orange, we examined the expression levels of all the TFs. The TFs that exhibited differential expression in response to cold stress at either 12 h or 24 h were selected for further analysis. A total of 530 differentially expressed TFs were identified. Four of these TFs are newly identified (**Supplementary Figure 6**, **Supplementary Table 7**). The five most prevalent transcription factor (TF) families (**Figure 5**) included ERF TFs. Most cold-responsive TFs were ERFs followed by MYB TFs. The expression of the majority of the *ERF* genes was elevated during cold stress, which provides evidence that ERFs. TFs play a pivotal role in cold tolerance in trifoliate orange. In contrast, the expression of a large number of *MYB* and *bHLH* families were downregulated after 24 h of cold stress compared to after 12 h of cold stress and to normal conditions In addition, 73 TFs were found to have undergone AS in response to cold stress (**Supplementary Table 8**). For example, *Pt8g001560* (a number of bZIP family) was differentially spliced in response to cold stress (**Supplementary Figure 7**). These observations indicate that both transcriptional and post-transcriptional regulation play pivotal roles in the response of trifoliate orange to cold stress.

## Discussion

In this study, we constructed a comprehensive full-length transcriptome for *Citrus trifoliata* by combining Iso-seq and RNA-seq. A total of 58,485 high-quality transcripts were generated with longer mean lengths than in the previously annotated genome (Huang et al., 2021). Additionally, some novel gene loci were identified by comparing the transcript structure to the previous annotation. It is widely acknowledged that Illumina sequencing offers significant advantages due to its cost-effectiveness, accuracy and rapid sequencing speed. Nevertheless, the shorter lengths of the Illumina sequence reads represent a limitation. In contrast, third-generation sequencing produces longer reads with higher error rates. Consequently, an increasing number of studies combine RNA-seq and Iso-seq technology to construct more comprehensive and higher-quality transcriptomes. Examples include studies done with sorghum (Abdel-Ghany et al., 2016), cotton (Wang et al., 2018a), rice (Zhang et al., 2019; Zhong et al., 2024), and maize (Wang et al., 2018b). In strawberry and pineapple (Liu et al., 2021; Feng et al., 2024), the researchers utilized data generated using Iso-seq and RNA-seq data and also manually revised annotations for each gene structure. This method produced high-quality annotations for the corresponding genomes that were not based solely on RNA-seq resources. In conclusion, our data provide the first comprehensive transcriptome analysis of a citrus genome, which will prove invaluable for the correction of structural errors in the annotation of genes.

Cold stress significantly impairs the growth and production of citrus (Dahro et al., 2023). Trifoliate orange is a highly cold-tolerant member of the citrus family. An in-depth understanding of the molecular underpinnings of cold tolerance in trifoliate orange will lead to the breeding of more cold tolerant varieties of citrus. The accumulation of substances that influence osmotic potential, including soluble sugars and proteins, is crucial for plants to tolerate cold stress. For example, INV has been demonstrated to enhance cold tolerance in citrus by catalyzing the catabolism of sucrose (Dahro et al., 2022). The expression of *INV* is regulated by AHL17 and *AHL14*. In tea plants, the expression of *SWEET17* was activated by *LHY*, which positively regulates cold tolerance (Wu et al., 2024). More recently, the influence of CBF-dependent pathways on *Tonoplast Sugar Transporter* (*TST*) was found to be critical for the accumulation of sugar in apples grown in low temperature conditions (Li et al., 2024). These studies demonstrate that the accumulation of soluble sugar contributes to cold tolerance in plants. In this study, we found that the expression of a number of genes associated with the accumulation of sugar was induced by cold stress, including *SWEETs*, *ERDs*, *INV2*, *SS* and *SPS1*. GC-MS revealed that the levels of three main soluble sugars were increased in response to cold stress and that this possibly contributes to cold tolerance in trifoliate orange. The candidate genes identified here provide important clues that will contribute to the dissection of the cold response network in citrus.

The regulation of the transcriptome and AS in citrus in response to cold stress were investigated utilizing hybrid sequencing. We found minimal overlap between the two data sets. Moreover, KEGG enrichment analysis indicated that in response to cold, transcriptional and AS regulation influence different biological processes. Our findings provide evidence to support the proposition that transcriptional and AS regulation are two parallel processes that function independently when plants experience cold stress, which is consistent with previous work (Liu et al., 2018; Wang et al., 2019). Furthermore, we found that the number of DSGs was relatively stable relative to DEGs. Indeed, 77.8% of the genes that were differentially spliced at 12 h were also differentially spliced at 24 h of cold stress. In contrast, only 40.6% of the genes were expressed at different levels after 12 h of cold stress and only 32.4% of the genes were differentially expressed after 24 h of cold stress. The KEGG enrichment analysis revealed that DEGs were enriched in signaling pathways, such as “MAPK signaling pathway”, or some pathways involved in the biosynthesis of metabolites that promote cold-tolerance, such as “starch and sucrose metabolism” and “alpha-linolenic acid metabolism”. In contrast, the DSGs were mainly enriched in genes associated with basic biological metabolic processes including “photosynthesis” and “carbon metabolism”. Collectively, our results demonstrate that the regulation of AS is independent or at least partially independent of transcriptional regulation.

It is well documented that elevated levels of reactive oxygen species (ROS) are toxic to plant cells because they damage nucleic acids and induce lipid peroxidation (Dreyer and Dietz, 2018; Huang et al., 2010). Therefore, the accumulation of ROS has a major influence on cell viability when plants are grown in abiotic stress conditions. Under normal conditions, a delicate equilibrium exists between ROS production and scavenging. However, this equilibrium can be disrupted by abiotic stresses, resulting in a rise in intracellular ROS levels. Antioxidant enzymes (APX, POD, SOD, CAT) play pivotal roles in the scavenging of ROS and the protection of plants from oxidative damage. In this study, we noticed that the expression of 12 *POD* and 4 *SOD* genes was induced in plants subjected to cold stress. The results demonstrated that the trifoliate orange possesses a robust capacity for the detoxification of ROS when grown in cold stress conditions, which may contribute to its cold tolerance.

Transcription factors (TFs) are critical in regulating the response to cold conditions by influencing the expression of specific functional genes. CBFs have been identified as key transcription factors involved in cold-responsive gene expression and are conserved in different plant species (Kidokoro et al., 2022). In recent years, some other TF families were reported to play key roles in cold tolerance in citrus, including ERFs (Khan et al., 2021; Zhang Y. et al., 2022) and bHLHs (Huang et al., 2013; Geng and Liu, 2018; Ming et al., 2021). In this study, we identified some genes encoding TFs that were expressed at markedly elevated levels in response to cold stress, including gene families that encode TFs belonging to the ERF, MYB, NAC, bHLH, and WRKY superfamilies. The majority of these TFs have yet to be elucidated with regard to their functions and target genes. Therefore, the cold-responsive TFs identified in this study represent a valuable addition to the collection of citrus TFs, providing a rich resource for the investigation of the transcriptional regulatory networks of plants.

## Conclusions

We performed a comprehensive investigation into the influence of cold stress on *C. trifoliata*, elucidating substantial physiological and molecular alterations. The findings of the study demonstrate that exposure to cold stress results in damage to cells and a decline in photosynthetic activity. In particular, we found an increase in electrolyte leakage, a decline in the maximum quantum efficiency of photosystem II (*F_v_*/*F_m_*), and an accumulation of reactive oxygen species (ROS). An analysis of the full-length transcriptome that utilized PacBio sequencing revealed that substantial alteration in gene expression and AS are major components of the cold stress response in *C. trifoliata*. These findings provide valuable insights into the regulation of transcription and AS that contribute to cold tolerance in *C. trifoliata*. This transcriptome and AS profiling constitute an invaluable resource for the functional evaluation of cold-responsive genes in *C. trifoliata*, which are presumed to possess considerable potential for the genetic engineering of cold tolerance in citrus.

## Data Availability

The datasets presented in this study can be found in online repositories. The names of the repository/repositories and accession number(s) can be found in the article/**Supplementary Material**.

## References

[B1] Abdel-Ghany S. E. Hamilton M. Jacobi J. L. Ngam P. Devitt N. Schilkey F. P.. (2016). A survey of the sorghum transcriptome using single-molecule long reads. Nat. Commun. 7, 11706. doi: 10.1038/ncomms11706 27339290 PMC4931028

[B2] Chen S. Zhou Y. Chen Y. Gu J. (2018). fastp: An ultra-fast all-in-one FASTQ preprocessor. Bioinformatics 34, i884–i890. doi: 10.1093/bioinformatics/bty560 30423086 PMC6129281

[B3] Chen C. Chen H. Zhang Y. Thomas H. R. Frank M. H. He Y.. (2020). TBtools: an integrative toolkit developed for interactive analyses of big biological data. Mol. Plant 13, 1194–1202. doi: 10.1016/j.molp.2020.06.009 32585190

[B4] Dahro B. Li C. Liu J. H. (2023). Overlapping responses to multiple abiotic stresses in citrus: from mechanism understanding to genetic improvement. Hortic. Adv. 1, 4. doi: 10.1007/s44281-023-00007-2

[B5] Dahro B. Wang Y. Khan M. Zhang Y. Fang T. Ming R.. (2022). Two AT-Hook proteins regulate A/NINV7 expression to modulate sucrose catabolism for cold tolerance in *Poncirus trifoliata* . New Phytol. 235, 2331–2349. doi: 10.1111/nph.v235.6 35695205

[B6] Dahro B. Wang F. Peng T. Liu J. H. (2016). PtrA/NINV, an alkaline/neutral invertase gene of *Poncirus trifoliata*, confers enhanced tolerance to multiple abiotic stresses by modulating ROS levels and maintaining photosynthetic efficiency. BMC Plant Biol. 16, 76. doi: 10.1186/s12870-016-0761-0 27025596 PMC4812658

[B7] Ding Y. Yang S. (2022). Surviving and thriving: How plants perceive and respond to temperature stress. Dev. Cell. 57, 947–958. doi: 10.1016/j.devcel.2022.03.010 35417676

[B8] Dreyer A. Dietz K. J. (2018). Reactive oxygen species and the redox-regulatory network in cold stress acclimation. Antioxidants 7, 169. doi: 10.3390/antiox7110169 30469375 PMC6262571

[B9] Feng J. Zhang W. Chen C. Liang Y. Li T. Wu Y.. (2024). The pineapple reference genome: Telomere-to-telomere assembly, manually curated annotation, and comparative analysis. J. Integr. Plant Biol. 66, 2208–2225. doi: 10.1111/jipb.13748 39109967

[B10] Florea L. Song L. Salzberg S. L. (2013). Thousands of exon skipping events differentiate among splicing patterns in sixteen human tissues. F1000Res 2, 188. doi: 10.12688/f1000research 24555089 PMC3892928

[B11] Geng J. Liu J. H. (2018). The transcription factor CsbHLH18 of sweet orange functions in modulation of cold tolerance and homeostasis of reactive oxygen species by regulating the antioxidant gene. J. Exp. Bot. 69, 2677–2692. doi: 10.1093/jxb/ery065 29474667 PMC5920331

[B12] Gordon S. P. Tseng E. Salamov A. Zhang J. Meng X. Zhao Z.. (2015). Widespread polycistronic transcripts in fungi revealed by single-molecule mRNA sequencing. PLoS ONE 10, e0132628. doi: 10.1371/journal.pone.0132628 26177194 PMC4503453

[B13] Huang X. S. Liu J. H. Chen X. J. (2010). Overexpression of PtrABF gene, a bZIP transcription factor isolated from *Poncirus trifoliata*, enhances dehydration and drought tolerance in tobacco via scavenging ROS and modulating expression of stress-responsive genes. BMC Plant Biol. 10, 230. doi: 10.1186/1471-2229-10-230 20973995 PMC3017851

[B14] Huang Y. Si Y. Dane F. (2011). Impact of grafting on cold responsive gene expression in Satsuma mandarin (*Citrus unshiu*). Euphytica 177, 25–32. doi: 10.1007/s10681-010-0243-7

[B15] Huang X. S. Wang W. Zhang Q. Liu J. H. (2013). A basic helix-loop-helix transcription factor, PtrbHLH, of *Poncirus trifoliata* confers cold tolerance and modulates peroxidase-mediated scavenging of hydrogen peroxide. Plant Physiol. 162, 1178–1194. doi: 10.1104/pp.112.210740 23624854 PMC3668048

[B16] Huang Y. Xu Y. Jiang X. Yu H. Jia H. Tan C.. (2021). Genome of a citrus rootstock and global DNA demethylation caused by heterografting. Horticult. Res. 8 (1), 69. doi: 10.1038/s41438-021-00505-2 PMC8012640 33790260

[B17] Huertas R. Catalá R. Jiménez-Gómez J. M. Mar Castellano M. Crevillén P. Piñeiro M.. (2019). Arabidopsis SME1 regulates plant development and response to abiotic stress by determining spliceosome activity specificity. Plant Cell. 31, 537–554. doi: 10.1105/tpc.18.00689 30696706 PMC6447010

[B18] Kalsotra A. Cooper T. A. (2011). Functional consequences of developmentally regulated alternative splicing. Nat. Rev. Genet. 12, 715–729. doi: 10.1038/nrg3052 21921927 PMC3321218

[B19] Khan M. Hu J. Dahro B. Ming R. Zhang Y. Wang Y.. (2021). ERF108 from *Poncirus trifoliata* (L.) Raf. functions in cold tolerance by modulating raffinose synthesis through transcriptional regulation of PtrRafS. Plant J. 108, 705–724. doi: 10.1111/tpj.v108.3 34398993

[B20] Kidokoro S. Shinozaki K. Yamaguchi-Shinozaki K. (2022). Transcriptional regulatory network of plant cold-stress responses. Trends Plant Sci. 27, 922–935. doi: 10.1016/j.tplants.2022.01.008 35210165

[B21] Kim D. Langmead B. Salzberg S. L. (2015). HISAT: A fast spliced aligner with low memory requirements. Nat. Methods 12, 357–360. doi: 10.1038/nmeth.3317 25751142 PMC4655817

[B22] Li Y. Mi X. Zhao S. Zhu J. Guo R. Xia X.. (2020b). Comprehensive profiling of alternative splicing landscape during cold acclimation in tea plant. BMC Genomics 21, 65. doi: 10.1186/s12864-020-6491-6 31959105 PMC6971990

[B23] Li B. Qu S. Kang J. Peng Y. Yang N. Ma B.. (2024). The MdCBF1/2-MdTST1/2 module regulates sugar accumulation in response to low temperature in apple. Plant J. 118, 787–801. doi: 10.1111/tpj.v118.3 38206080

[B24] Li S. Yu X. Cheng Z. Zeng C. Li W. Zhang L.. (2020a). Large-scale analysis of the cassava transcriptome reveals the impact of cold stress on alternative splicing. J. Exp. Bot. 71, 422–434. doi: 10.1093/jxb/erz444 31713628

[B25] Liao Y. Smyth G. K. Shi W. (2014). featureCounts: an efficient general purpose program for assigning sequence reads to genomic features. Bioinformatics 30, 923–930. doi: 10.1093/bioinformatics/btt656 24227677

[B26] Liu T. Li M. Liu Z. Ai X. Li Y. (2021). Reannotation of the cultivated strawberry genome and establishment of a strawberry genome database. Hortic. Res. 8, 41. doi: 10.1038/s41438-021-00476-4 33642572 PMC7917095

[B27] Liu Z. Qin J. Tian X. Xu S. Wang Y. Li H.. (2018). Global profiling of alternative splicing landscape responsive to drought, heat and their combination in wheat (Triticum asetivum L.). Plant Biotechnol. J. 16, 714–726. doi: 10.1111/pbi.2018.16.issue-3 28834352 PMC5814593

[B28] Livak K. J. Schmittgen T. D. (2001). Analysis of relative gene expression data using real-time quantitative PCR and the 2-ΔΔCT method. Methods 25, 402–408. 11846609 10.1006/meth.2001.1262

[B29] Love M. I. Huber W. Anders S. (2014). Moderated estimation of fold change and dispersion for RNA-seq data with DESeq. 2. Genome Biol. 15, 550. doi: 10.1186/s13059-014-0550-8 25516281 PMC4302049

[B30] Luo Y. Hu S. Zhang B. (2019). PacBio full-length cDNA sequencing integrated with RNA-seq reads drastically improves the discovery of splicing transcripts in rice. Plant J. 97, 296–305. 30288819 10.1111/tpj.14120

[B31] Ming R. Zhang Y. Wang Y. Khan M. Dahro B. Liu J. H. (2021). The JA-responsive MYC2-BADH-like transcriptional regulatory module in *Poncirus trifoliata* contributes to cold tolerance by modulation of glycine betaine biosynthesis. New Phytol. 229, 2730–2750. doi: 10.1111/nph.v229.5 33131086

[B32] Reddy A. S. Marquez Y. Kalyna M. Barta A. (2013). Complexity of the alternative splicing landscape in plants. Plant Cell. 25, 3657–3683. doi: 10.1105/tpc.113.117523 24179125 PMC3877793

[B33] Sahin-Çevik M. (2013). Identification and expression analysis of early cold-induced genes from cold-hardy Citrus relative Poncirus trifoliata (L.) Raf. Gene 512 (2), 536–545. doi: 10.1016/j.gene.2012.09.084 23026217

[B34] Salmela L. Rivals E. (2014). LoRDEC: accurate and efficient long read error correction. Bioinformatics 30, 3506–3514. doi: 10.1093/bioinformatics/btu538 25165095 PMC4253826

[B35] Shen S. Park J. W. Lu Z. X. Lin L. Henry M. D. Wu Y. N.. (2014). rMATS: robust and flexible detection of differential alternative splicing from replicate RNA-Seq data. Proc. Natl. Acad. Sci. U. S. A. 111, E5593–E5601. doi: 10.1073/pnas.1419161111 25480548 PMC4280593

[B36] Shi Y. Ding Y. Yang S. (2018). Molecular regulation of CBF signaling in cold acclimation. Trends Plant Sci. 23, 623–637. doi: 10.1016/j.tplants.2018.04.002 29735429

[B37] Talon M. Caruso M. Gmitter F. G. (2020). The Genus Citrus Vol. 16. Ed. Talon M. (Woodhead Publishing). doi: 10.1016/C2016-0-02375-6

[B38] Wang X. Chen S. Shi X. Liu D. Zhao P. Lu Y.. (2019). Hybrid sequencing reveals insight into heat sensing and signaling of bread wheat. Plant J. 98, 1015–1032. doi: 10.1111/tpj.2019.98.issue-6 30891832 PMC6850178

[B39] Wang B. Regulski M. Tseng E. Olson A. Goodwin S. McCombie W. R.. (2018b). A comparative transcriptional landscape of maize and sorghum obtained by single-molecule sequencing. Genome Res. 28, 921–932. doi: 10.1101/gr.227462.117 29712755 PMC5991521

[B40] Wang T. Wang H. Cai D. Gao Y. Zhang H. Wang Y.. (2017). Comprehensive profiling of rhizome-associated alternative splicing and alternative polyadenylation in moso bamboo (Phyllostachys edulis). Plant J. 91, 684–699. doi: 10.1111/tpj.2017.91.issue-4 28493303

[B41] Wang M. Wang P. Liang F. Ye Z. Li J. Shen C.. (2018a). A global survey of alternative splicing in allopolyploid cotton: landscape, complexity and regulation. New Phytol. 217, 163–178. doi: 10.1111/nph.2018.217.issue-1 28892169

[B42] Wu Y. Di T. Wu Z. Peng J. Wang J. Zhang K.. (2024). CsLHY positively regulates cold tolerance by activating CsSWEET17 in tea plants. Plant Physiol. Biochem. 207, 108341. doi: 10.1016/j.plaphy.2024.108341 38266557

[B43] Wu T. D. Watanabe C. K. (2015). GMAP: a genomic mapping and alignment program for mRNA and EST sequences. Bioinformatics 1, 1859–1875. doi: 10.1093/bioinformatics/bti310 15728110

[B44] Yu K. Xu Q. Da X. Guo F. Ding Y. Deng X. X. (2012). Transcriptome changes during fruit development and ripening of sweet orange (Citrus sinensis). BMC Genomics 13, 10. doi: 10.1186/1471-2164-13-10 22230690 PMC3267696

[B45] Zhang S. Liang M. Wang N. Xu Q. Deng X. Chai L. (2018). Reproduction in woody perennial Citrus: an update on nucellar embryony and self-incompatibility. Plant Reprod. 31, 43–57. doi: 10.1007/s00497-018-0327-4 29457194

[B46] Zhang J. Liang Y. Zhang S. Xu Q. Di H. Zhang L.. (2022). Global landscape of alternative splicing in maize response to low temperature. J. Agric. Food Chem. 70, 15715–15725. doi: 10.1021/acs.jafc.2c05969 36479939

[B47] Zhang Y. Ming R. Khan M. Wang Y. Dahro B. Xiao W.. (2022). ERF9 of *Poncirus trifoliata* (L.) Raf. undergoes feedback regulation by ethylene and modulates cold tolerance via regulating a glutathione S-transferase U17 gene. Plant Biotechnol. J. 20, 183–200. doi: 10.1111/pbi.13705 34510677 PMC8710834

[B48] Zhang G. Sun M. Wang J. Lei M. Li C. Zhao D.. (2019). PacBio full-length cDNA sequencing integrated with RNA-seq reads drastically improves the discovery of splicing transcripts in rice. Plant J. 97, 296–305. doi: 10.1111/tpj.2019.97.issue-2 30288819

[B49] Zhong Y. Luo Y. Sun J. Qin X. Gan P. Zhou Z.. (2024). Pan-transcriptomic analysis reveals alternative splicing control of cold tolerance in rice. Plant Cell. 36, 2117–2139. doi: 10.1093/plcell/koae039 38345423 PMC11132889

