# Supplementary material for: Full-length single-molecule sequencing uncovers novel insight into the global landscape of the cold stress response in trifoliate orange (Citrus trifoliata)
Source: Front Plant Sci. 2024 Nov 18;15:1506414. doi: 10.3389/fpls.2024.1506414 (PMC11608947; doi:10.3389/fpls.2024.1506414)
Supplement: Supplementary file 1 [file DataSheet1.docx]

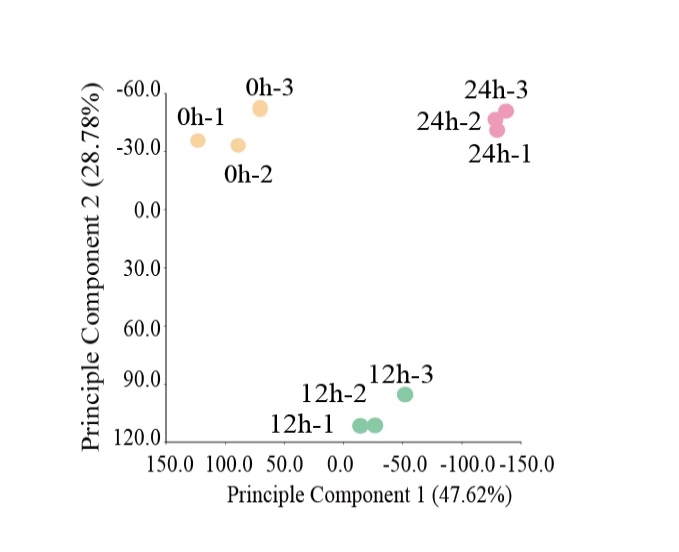


Figure S1. PCA analysis of the RNA-seq samples.


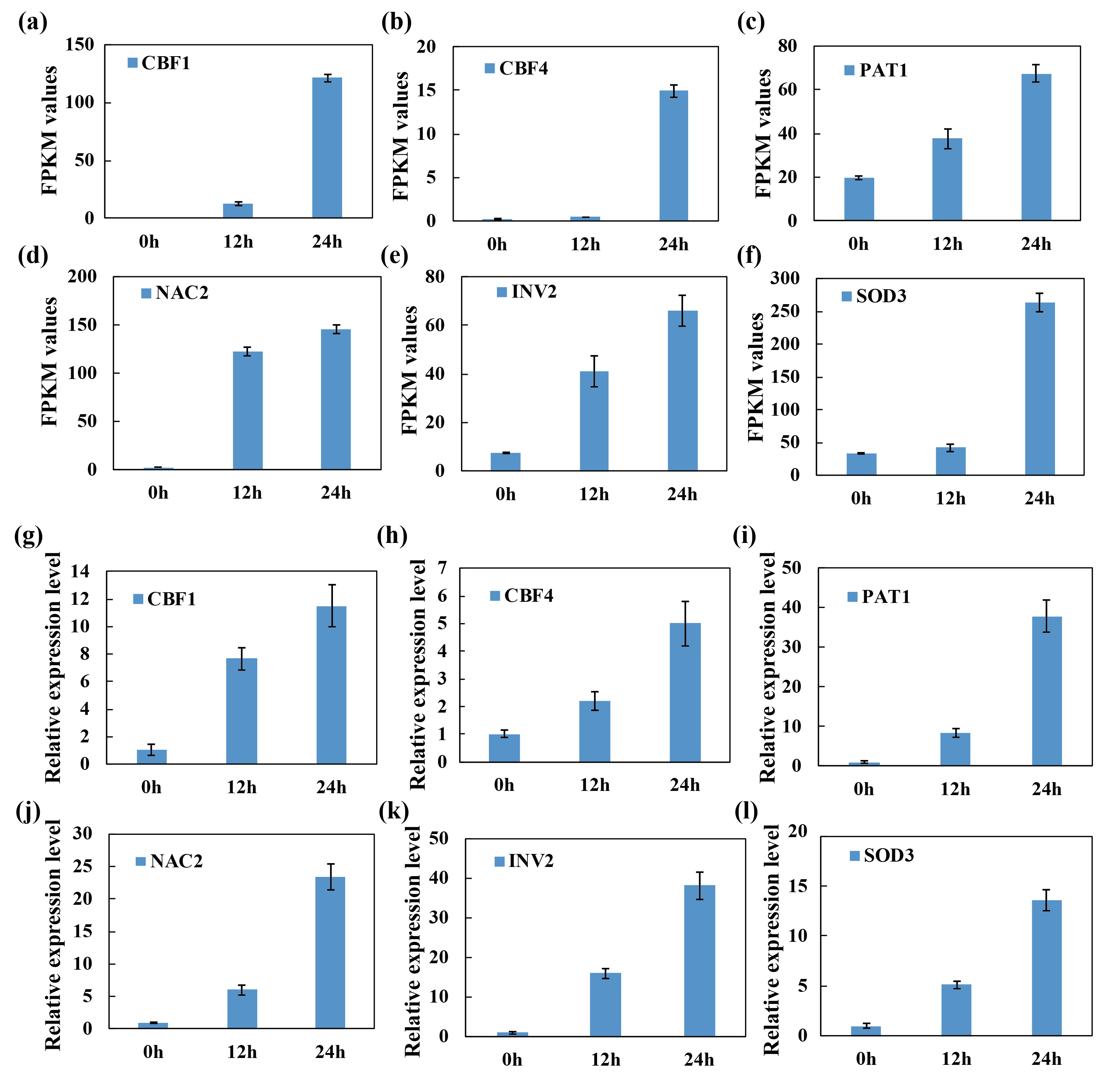


Figure S2. Differentially expressed genes (DEGs) in trifoliate orange plants in response to cold stress valeted by RNA-seq and RT-qPCR.


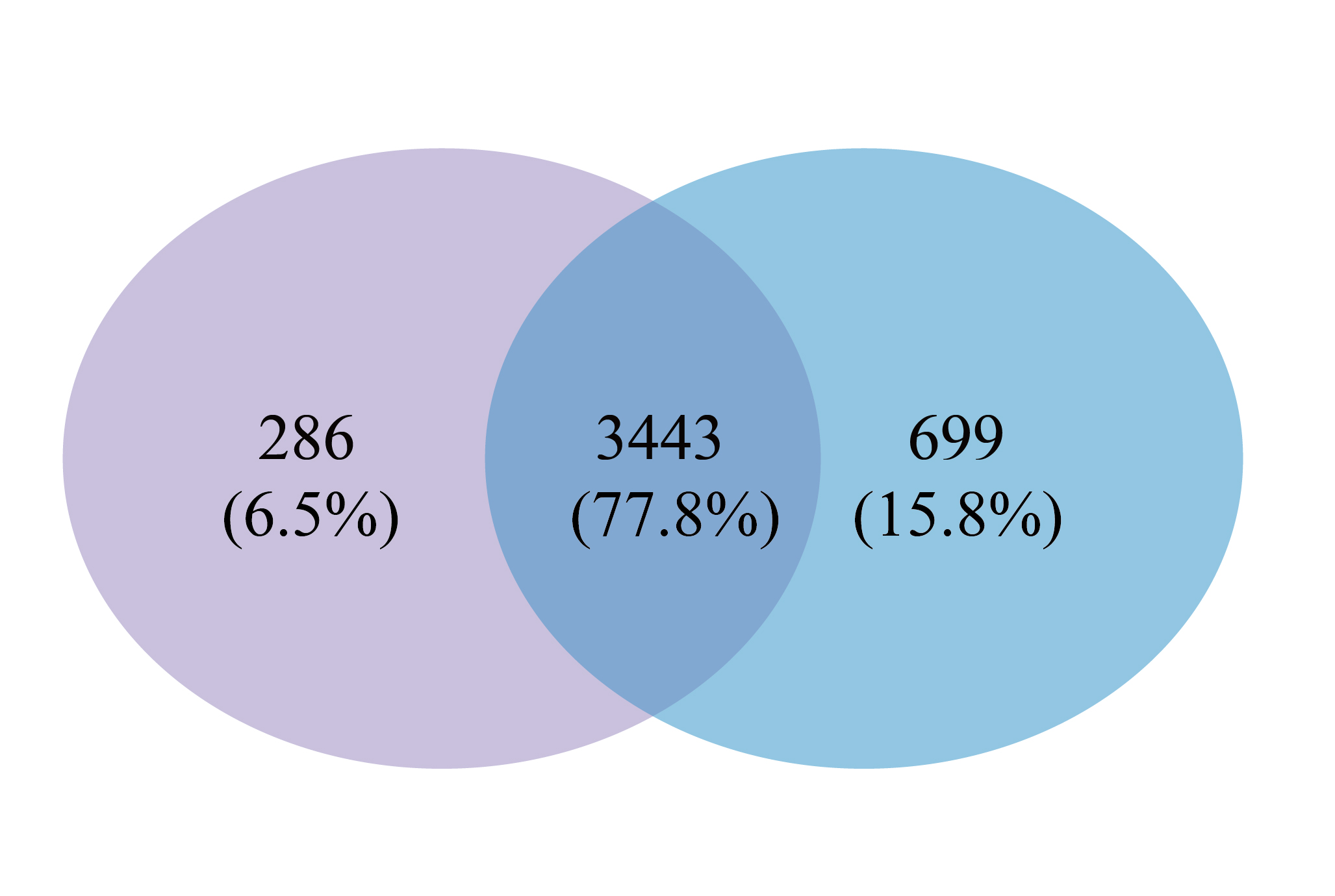


Figure S3. Venn diagram indicated the commonly DSGs between cold stress for 12h and 24h.


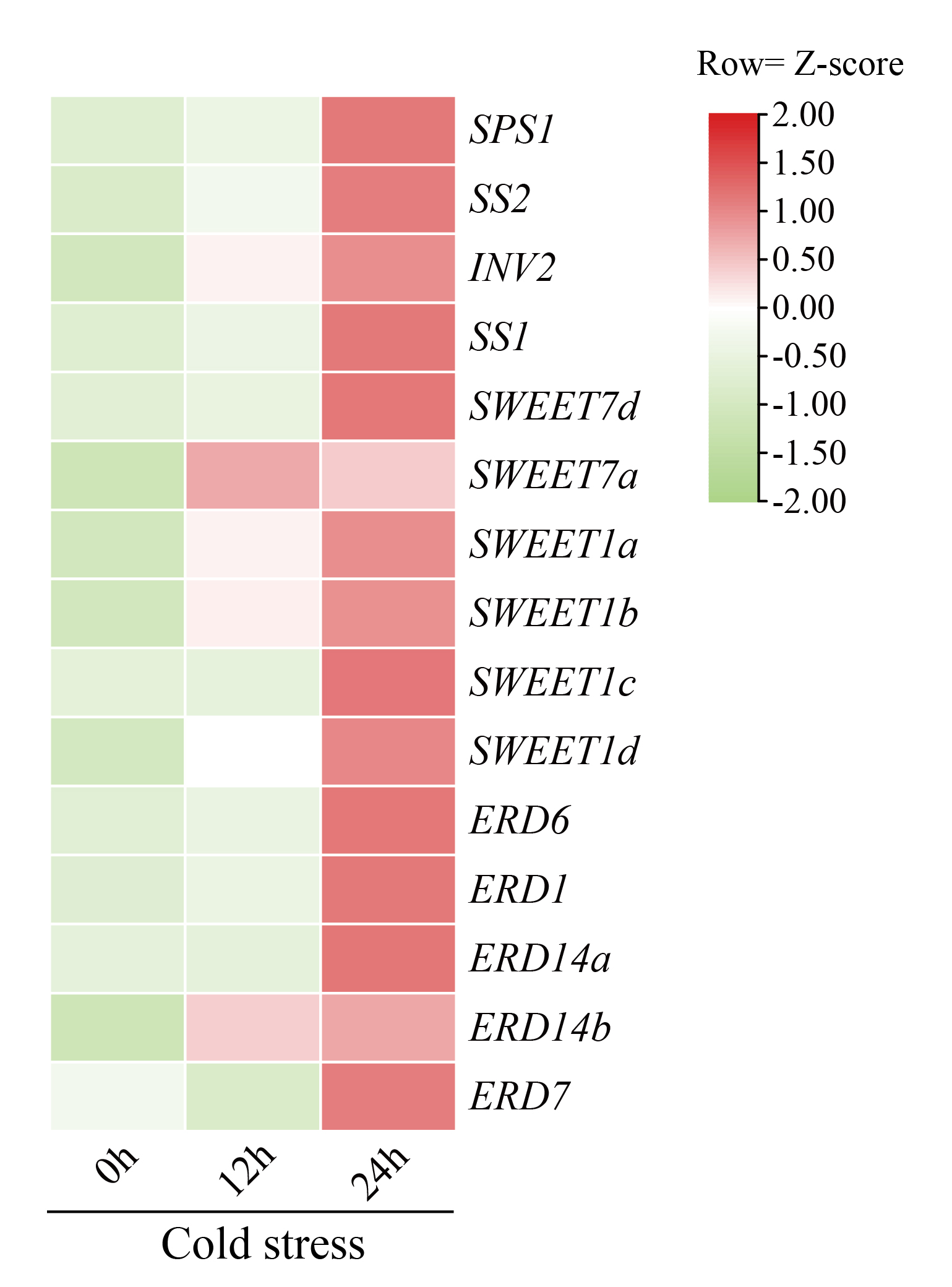


Figure S4. The expression level of up-regulated genes involved in sugar metabolism showed by heatmap.


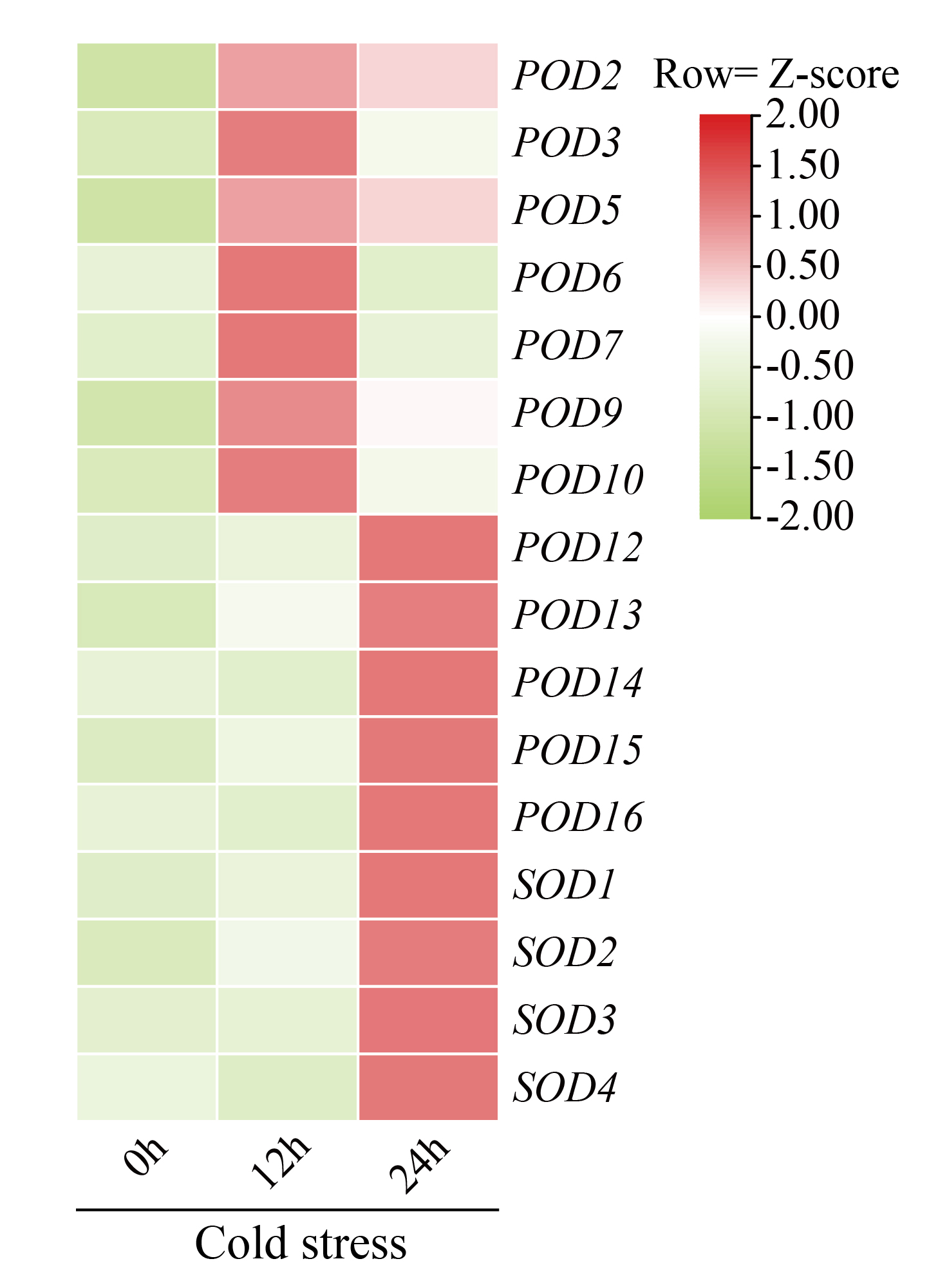


Figure S5. The expression level of up-regulated genes involved ROS scavenging showed by heatmap.


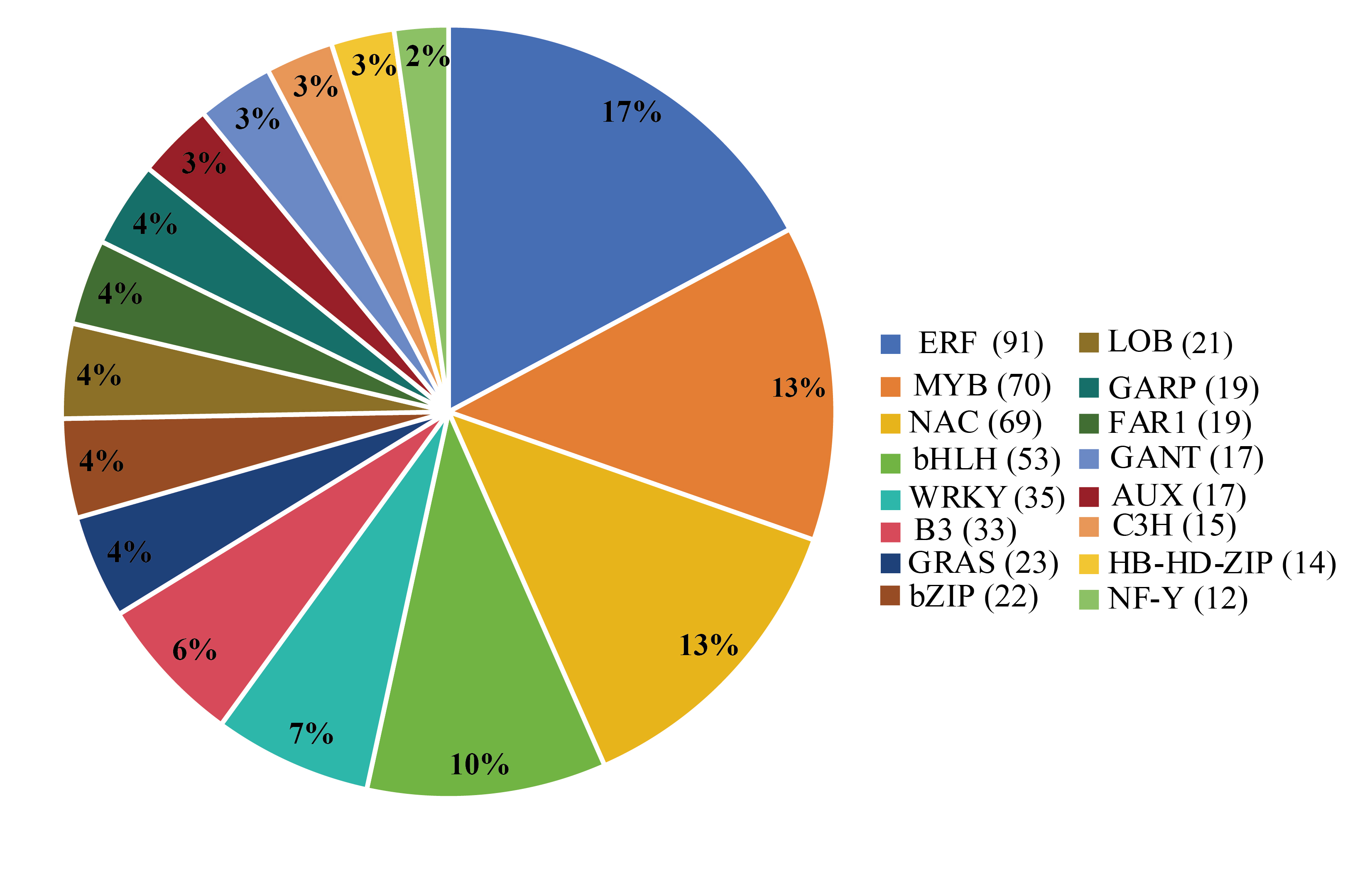


Figure S6. Proportion and number of differentially expressed transcription factors.


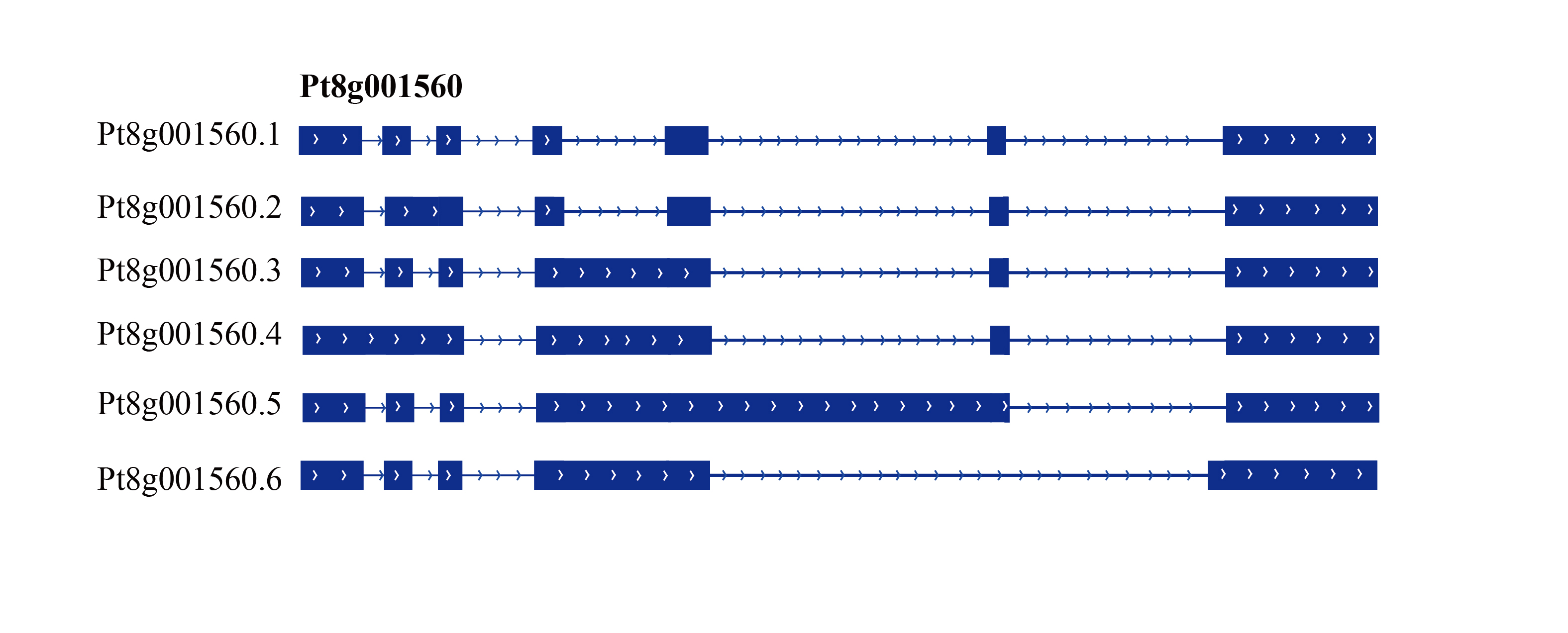
Figure S7. Schematic representation of the isoforms produced by *Pt8g001560* (a number a bZIP family).
